# Preoperative laboratory evaluation of patients aged over 40 years undergoing elective non-cardiac surgery

**DOI:** 10.1590/S1516-31802005000200003

**Published:** 2005-03-02

**Authors:** Fábio Yoshito Ajimura, Alex Silva Santos Freire Maia, Adriana Hachiya, Alexandra Sayuri Watanabe, Maria do Patrocínio Tenório Nunes, Mílton de Arruda Martins, Fábio Santana Machado

**Keywords:** Preoperative care, Blood tests, Routine diagnostic tests, Intraoperative complications, Laboratory techniques and procedures, Cuidados pré-operatórios, Testes hematológicos, Testes diagnósticos de rotina, Complicações intra-operatórias, Técnicas e procedimentos de laboratório

## Abstract

**CONTEXT AND OBJECTIVE:**

Although it is generally agreed that a medical history and physical examination should be obtained as part of preoperative evaluation, there is still substantial controversy about the additional benefi ts of preoperative screening tests. The objective of the present study was to determine the percentage of abnormalities on laboratory tests among a population that underwent non-cardiac surgery and to correlate these tests with changes in preoperative evaluation management.

**DESIGN AND SETTING:**

Cross-sectional study, carried out in a University Hospital.

**METHODS:**

991 patients aged over 40 years undergoing elective non-cardiac surgery from July 1997 to January 2000 were studied. Blood cell count, serum sodium, potassium, urea and creatinine, prothrombin, thrombin and partial thromboplastin time, electrocardiogram and chest X-ray were evaluated.

**RESULTS:**

Out of the 957 electrocardiograms performed, some type of abnormality was found in 504 cases (50.9%) and, among the 646 chest X-rays requested, 271 (42.0%) displayed alterations. Laboratory tests showed abnormal values ranging from 5.1% (prothrombin time) to 41.0% (hematocrit). Increased percentages of abnormal tests with increasing patient age were also observed.

**CONCLUSIONS:**

Although there were substantial numbers of screening test abnormalities in preoperative evaluations, these results seldom interfered in patient management.

## RESUMO

**CONTEXTO E OBJETIVO:**

Embora seja consenso que uma anamnese e um exame clínico devam ser realizados como parte da avaliação pré-operatória, ainda há grande controvérsia a respeito dos benefícios adicionais de exames subsidiários de rastreamento na avaliação pré-operatória. Neste estudo objetivamos determinar a porcentagem de exames subsidiários alterados em uma população que foi submetida a cirurgia não-cardíaca e correlacionamos as anormalidades observadas nestes exames com mudanças realizadas nas condutas pré-operatórias.

**TIPO DE ESTUDO E LOCAL:**

Estudo transversal desenvolvido em Hospital Universitário.

**MÉTODOS:**

991 pacientes com mais de 40 anos, que foram submetidos a operações não-cardíacas eletivas de julho de 1997 a janeiro de 2000 foram estudados. Foram avaliados o hemograma, as dosagens séricas de sódio, potássio, uréia e creatinina, os tempos de protrombina, trombina e tromboplastina parcial ativada, o eletrocardiograma e a radiografia de tórax.

**RESULTADOS:**

Dentre os 957 eletrocardiogramas realizados, alguma alteração foi observada em 504 (50,9%) casos e, dentre as 646 radiografias de tórax feitas, 271 (42,0%) apresentaram alterações. As porcentagens de exames de laboratório alterados variaram de 5,1% (tempo de protrombina) a 41,0% (hematócrito). Observamos, também, um aumento na porcentagem de exames alterados em pacientes mais idosos.

**CONCLUSÕES:**

Embora um grande número de exames subsidiários alterados seja observado na avaliação pré-operatória, estes resultados raramente interferem na conduta dos pacientes.

## INTRODUCTION

The objective of preoperative evaluation is to reduce perioperative morbidity and mortality.^1^ Preoperative medical evaluation is needed to assess individual patients’ risks of perioperative morbidity and mortality. Laboratory tests are requested with the intention of obtaining more information about the patient who will be submitted to surgery, so as to optimize his/her clinical condition before the procedure and reduce intra and postoperative complications. Nevertheless, routine use of large numbers of screening tests may substantially increase the cost of perioperative care.^2^

Laboratory tests may be ordered to evaluate problems presented, monitor known abnormalities or detect unsuspected disease. A wide variety of routine screening tests have been used for preoperative assessment. These includes clinical assays,^3,4^ prothrombin, thrombin and partial thromboplastin times,^5,6^ urinalysis,^7^ electrocardiogram^8^ and plain chest radiography.^9^ However, there is a lot of controversy about routine laboratory evaluation of preoperative patients. Several studies have affirmed that prior clinical conditions are correlated with the risk of complications during the intra and postoperative periods.^10,11^ Screening tests detect abnormalities that are clinically unimportant for the scheduled surgery, and they are usually ignored by clinicians. Abnormalities that are clinically important can usually be predicted from a complete history and physical examination.

In addition, unnecessary testing may cause harm to the patient due to overtreatment of borderline or false-positive results. In this respect, the indiscriminate use of such laboratory exams remains a matter for discussion, since costs may be increased without reducing perioperative complications.^12^

In this study, we determined the prevalence of laboratory test abnormalities among a population submitted to non-cardiac surgery and correlated these with changes in preoperative evaluation management.

## METHODS

All patients aged over 40 years undergoing elective non-cardiac surgery that had been submitted to preoperative clinical evaluation in the Division of General Internal Medicine of Hospital das Clínicas of the School of Medicine of the Universidade de São Paulo between July 1997 and January 2000 were included in the study. Informed consent was obtained. The tests performed in this study were blood assays (sodium and potassium), serum urea and creatinine, coagulation tests (prothrombin, thrombin and partial thromboplastin times), red and white blood cell and platelet counts, and hemoglobin and hematocrit determination. Each result was classified as normal or abnormal, according to reference normal values.

We also analyzed electrocardiograms and chest X-rays. The electrocardiograms were evaluated either by a cardiologist or a senior internal medicine resident. Radiological examinations were evaluated by a radiologist or a senior internal medicine resident.

## RESULTS

There were 541 men (54.6%) and 450 women (45.4%) in the study. The mean age was 63.6 ± 11.0 years.

Out of the 957 electrocardiograms performed, some type of abnormality was found in 504 cases (52.7%) (Table 1). The most common alterations observed were abnormal t-wave (19.1%), signs of left ventricular hypertrophy (10.3%), anterior left-bundle hemiblock (5.8%), q-waves (5.2%), left atrial hypertrophy (4.4%) and right bundle branch block (4.3%).

**Table 1:** Preoperative most common electrocardiogram results among 991 patients operated in São Paulo, Brazil

Type	Number	(%)
Normal	453	47.3
Abnormal t-wave	182	19.1
Left ventricular hypertrophy	99	10.3
Anterior left-bundle hemiblock	56	5.8
Q-wave	50	5.2
Left atrial hypertrophy	42	4.4
Right bundle branch block	41	4.3

*Total of 957 electrocardiograms performed.*

Even patients whose risk level changed (8.7%), according to the preoperative evaluation criteria of the American College of Physicians,^13^ did not have their surgery suspended. Rather, there was a more detailed evaluation.

We found 271 cases of abnormal chest X-rays among the 646 that were performed (41.7%), and their specific alterations can be seen in Table 2. Twenty-three percent of the X-rays showed signs suggestive of cardiomegaly and 11.5% presented signs that suggested the presence of chronic pulmonary disease.

**Table 2:** Preoperative chest X-ray abnormalities among 991 patients operated in São Paulo, Brazil

Type	Number	(%)
Normal	383	59.3
Cardiomegaly	149	23.1
Chronic pulmonary disease signs*	74	11.5
Others	48	7.5

*Total number of chest X-rays performed was 646: not all patients underwent X-ray examination;*

* *hyperinflation, abnormal diaphragm curvature, bullae and airway wall thickening.*

The specific abnormalities in each laboratory test are described in Table 3.

**Table 3:** Preoperative laboratory test results among 991 patients operated in São Paulo, Brazil

Test	Mean ± SD	% outside reference values	% above reference values	% below reference values
Serum sodium	139.7 ± 4.0 (mEq/l)	11.3	2.0	9.3
Serum potassium	4.4 ± 0.5 (mEq/l)	13.6	10.1	3.5
Blood urea	39.9 ± 18.5 (mg/dl)	25.3	25.3	0.0
Serum creatinine	1.1 ± 0.5 (mg/dl)	13.2	10.9	2.3
Prothrombin time	11.5 ± 1.6 (sec)	5.1	3.0	2.1
Platelet count	260,249 ± 90,154 (units/mm3)	13.1	6.9	6.2
Hematocrit	40.0 ± 5.2 (%)	41.0	1.2	39.9
White blood cells	8.2 ± 2.9 (units x 103/mm3)	31.4	22.0	9.4

*SD = standard deviation.*

The most common alterations verified were in relation to hematocrit (41%), white blood cells (31.4%), blood urea (25.3%), and altered results were also found in potassium (13.6%), creatinine (13.2%), platelet count (13.1%), sodium (11.3%) and prothrombin time (5.1%).

We also verified increased percentages of abnormal laboratory tests, abnormal chest X-ray and electrocardiogram with increasing patient age (Table 4). In all the cases of abnormal screening test values, we investigated whether this abnormal laboratory test, X-ray or electrocardiogram resulted in a change in the conduct adopted by the attending physicians or the surgeon. We did not observe any major change in their conduct due to the screening tests results.

**Table 4:** Percentage of abnormal preoperative screening tests, according to patients’ ages among 991 patients operated in São Paulo, Brazil

Age (years)	Hematocrit (%)	Blood urea (mg/dl)	Serum creatinine (mg/dl)	Abnormal electrocardiogram	Abnormal chest X-ray
40-60	37.4	16.2	10.0	43.1	33.3
> 60	43.4*	31.1*	15.0*	58.6*	45.8*

* *p < 0.05 compared to percentage obtained for patients aged 40-60 years.*

## DISCUSSION

It is known that high routine use of laboratory tests is frequently made and that abnormalities are often only noticed in such tests and not during physical examination, when a preoperative evaluation is performed. Regardless of whether such tests are performed, patient management usually remains unaltered. It is very difficult to establish the prognostic meaning of these abnormalities observed in the laboratory. Reducing the number of laboratory tests will result in decreased costs, consultation time and psychological stress associated with false-positive results.^14^

Delahunt and Turnbull^15^ studied 860 patients who were to be submitted to elective surgery, and found 172 abnormal examinations (20%). In none of these cases was there any change in the management procedure. In another study, Muskett and McGreevy^16^ found 477 tests (35.3%) with abnormal results. However, in only 76 (5.9%) was there a change of conduct. This hypothesis that few interventions are made strictly as a result of abnormal laboratory results has also been verified in other studies.^16,17^

We observed abnormalities in laboratory tests, but in none of the patients studied was there a change in management due to the abnormal values observed. According to the literature, although patients with a history of renal impairment may require changes to the type and dose of anesthetics and adjuvant medications administered,^18^ it is not clear that blood assay screening must be performed to find unsuspected abnormalities among patients without any history of renal disease. The majority of unsuspected abnormalities found through laboratory tests are observed among the elderly.^19^ This concords with our study, in which we found a greater percentage of abnormalities among patients aged over 60 years.

Our results from hematological tests, which are usually performed to prevent bleeding and clotting disorders among surgical patients, showed that 5.1% of such patients presented abnormal prothrombin time, but in none of these cases was there a change in patient management. Preoperative identification of individuals at high risk of bleeding during major elective surgery is obviously important. However, screening is expensive and may be inappropriate in low-risk groups. According to previous studies^20,21^ with similar results, preoperative assessment should be based on previous bleeding and, if the patient has no history of bleeding and physical examination is also negative, pre-operative screening for coagulation would be unnecessary.

We found a high percentage of abnormal chest X-rays, corresponding to 42.0% of the sample. This was the highest abnormality rate among all the preoperative tests studied. This percentage increased with age. Unanticipated abnormalities on chest X-rays among older adults are relatively common,^22^ whereas in younger populations they are rare.^23^ Nonetheless, the precise relationship between chest radiographic findings and perioperative morbidity has not been clarified.

Electrocardiograms have been considered by many physicians to be a very important preoperative screening test, because the presence of preoperative cardiac problems may result in significant postoperative morbidity. Electrocardiographic abnormalities were frequently found in our study. A routine electrocardiogram is recommended before elective non-cardiac surgery, for all patients aged over 40 years,^24,25^ because abnormalities are common, especially among elderly patients, and cardiac disease is related to significant perioperative morbidity.

Although we observed a high number of patients with some abnormalities in the screening tests performed, there was no change in the medical conduct or surgical procedure caused by these results. However, with regard to the electrocardiogram examination alone, 8.7% of the patients who underwent this test had a result that changed their risk level, according to the preoperative evaluation criteria of the American College of Physicians.^13^ In such cases, the surgical procedures were not suspended, but there was a more careful evaluation of the patient in order to optimize his/her clinical condition before and after the surgical procedure.

## CONCLUSIONS

A high percentage of abnormal laboratory examinations was observed among patients submitted to preoperative evaluation for non-cardiac surgery, and this percentage increased with age. Abnormal laboratory tests did not interfere with patient management. Abnormal electrocardiogram results changed the cardiac risk level in 8.7% of such patients, although these situations did not interfere with the surgical procedure.
